# Are the Recent Secular Increases in Waist Circumference among Children and Adolescents Independent of Changes in BMI?

**DOI:** 10.1371/journal.pone.0141056

**Published:** 2015-10-27

**Authors:** David S. Freedman, Brian K. Kit, Earl S. Ford

**Affiliations:** 1 Division of Nutrition, Physical Activity, and Obesity, Centers for Disease Control and Prevention, Atlanta, GA, United States of America; 2 Division of Health and Nutrition Examination Surveys, National Center for Health Statistics, Centers for Disease Control and Prevention, Hyattsville, MD, United States of America; 3 Division of Population Health, Centers for Disease Control and Prevention, Atlanta, GA, United States of America; Johns Hopkins Bloomberg School of Public Health, UNITED STATES

## Abstract

**Background:**

Several studies have shown that the waist circumference of children and adolescents has increased over the last 25 years. However, given the strong correlation between waist circumference and BMI, it is uncertain if the secular trends in waist circumference are independent of those in BMI.

**Methods:**

We analyzed data from 6- to 19-year-olds who participated in the 1988–1994 through 2011–2012 cycles of the National Health and Nutrition Examination Survey to assess whether the trends in waist circumference were independent of changes in BMI, race-ethnicity and age.

**Results:**

Mean, unadjusted levels of waist circumference increased by 3.7 cm (boys) and 6.0 cm (girls) from 1988–94 through 2011–12, while mean BMI levels increased by 1.1 kg/m^2^ (boys) and 1.6 kg/m^2^ (girls). Overall, the proportional changes in mean levels of both waist circumference and BMI were fairly similar among boys (5.3%, waist vs. 5.6%, BMI) and girls (8.7%, waist vs. 7.7%, BMI). As assessed by the area under the curve, adjustment for BMI reduced the secular increases in waist circumference by about 75% (boys) and 50% (girls) beyond that attributable to age and race-ethnicity. There was also a race-ethnicity interaction (p < 0.001). Adjustment for BMI reduced the secular trend in waist circumference among non-Hispanic (NH) black children (boys and girls) to a greater extent (about 90%) than among other children.

**Conclusions:**

Our results indicate that among children in the U.S., about 75% (boys) and 50% (girls) of the secular increases in waist circumference since 1988–94 can be accounted for by changes in BMI. The reasons for the larger independent effects among girls and among NH blacks are uncertain.

## Introduction

The prevalence of childhood obesity, defined as a BMI ≥ 95^th^ percentile of the CDC growth charts [1,2], among 6- to 19-year-olds in the U.S. increased from about 4.5% in the 1960s [3] to about 20% in 2011–12 [4]. Secular increases among 2- to 5-year-olds have been smaller, with the prevalence of obesity increasing from 5% in 1971–74 [3] to 12.1% in 2009–10, but then falling to 8.4% in 2011–12 [4]. The secular trends in obesity, among both children and adults, have slowed over the last 10 to 15 years [4,5].

Although BMI may be as strongly correlated with some metabolic complications as is body fatness [6–9], several consequences of obesity may be more strongly associated with the amount of abdominal (or visceral) fat [10]. For example, several investigators have found that the waist circumference (either alone or in conjunction with other body measures) of adults may be a better predictor of adverse health outcomes than is BMI [11–15]. Among children, several studies have found that waist circumference and BMI have very similar abilities to identify children who have an adverse cardio-metabolic risk profile [16,17]. The very strong correlation (r ~ 0.9) between BMI and waist circumference, among both children and adults, however, complicates the assessment of the independent effects of body fat distribution [18].

As is the case for adults [19–22], the waist circumference of children in several countries has increased in recent decades [19,20,23–25] and a recent review concluded that the secular increases in waist circumference have been larger than those for BMI [20]. These increases in abdominal obesity have been attributed to various factors, including changes in energy intake and physical activity, increased stress, and endocrine disruptors [26–28]. However, it is possible that much of the secular increase in waist circumference is no greater than what would be expected given the increases that have occurred in BMI. The objective of the current study is to assess the extent to which the recent trends in waist circumference among children are independent of changes in BMI.

## Methods

We used data from the National Health and Nutrition Examination Survey (NHANES) from 1998–94 (NHANES III) and from the 7 2-year cycles that have been conducted from 1999–2000 through 2011–2012 [29]. NHANES employs a multi-stage, stratified, cluster sampling design to select a representative sample of the US civilian, non-institutionalized population. The surveys were approved by the ethics review board, and parental permission was obtained for minors under the age of 18 years. 7- to 17-year-olds were also asked to provide documented assent, and consent was obtained for subjects who were ≥ 18 years of age. Age was calculated as age in months at the time of examination.

Preliminary analyses indicated that the secular trends in the body size measures were much greater among 6- to 19-year-olds than among 2- to 5-year-olds, these younger children are not included in the current analyses. We focus on 6- to 19-year-olds who had measurements of height, weight and waist circumference (n = 26,433). Race and ethnicity were self-reported, and for these analyses, participants are classified as non-Hispanic (NH) white, NH black, Mexican-American, or other (which includes Hispanics from other countries and persons who reported more than 1 race). The response rates varied by the year of survey cycle, but the unweighted response rates for the examined 6- to 19-year-olds varied from 84% to 86% in 1999–2000, while the comparable rates were 74% to 78% in 2011–12 [30].

During the NHANES physical examination, weight, height, and waist circumference were measured in a standardized fashion [31]. Body mass index (BMI) was calculated as weight (kg) divided by height (m)^**2**^. BMI-for-age z-scores and percentiles were calculated by expressing a child’s BMI relative to children of the same sex and age who participated in national studies conducted from 1963–65 to 1988–94 [32]. Obesity is defined as a BMI ≥ 95th percentile (for children of the same sex and age) of the CDC Growth Charts [2,32] or a BMI ≥ 30 kg/m^2^. Severe obesity is defined as a BMI ≥ 120% of the 95^th^ percentile in the CDC growth charts [33].

Waist circumference was measured to the nearest 1 mm just above the iliac crest using a steel measuring tape [31]. Although we primarily focus on waist circumference, some analyses examine secular trends in the waist to height ratio, a measure that has been proposed, in part, because levels of this ration remain fairly constant throughout childhood and adulthood [34].

### Statistical Methods

All analyses used the examination sample weights and accounted for the sample design using the *survey* package in R [35,36]. Mean levels of BMI, waist circumference, and other body size measures are shown by sex and survey cycle; confidence intervals for the estimated prevalences of obesity were calculated as suggested by Korn and Graubard [37]. To summarize the secular trends in waist circumference, we focused on an 8-level categorical variable for survey cycle, with 1988–1994 (NHANES III) serving as the reference category. Waist circumference was predicted by survey cycle, race-ethnicity and age in one regression model, and to examine whether differences across studies were independent of BMI, BMI was included as an additional predictor in a second model. All analyses controlled for race and age to exclude the possibility that changes in waist circumference over time were attributable to changes in these characteristics. Both BMI and age were modeled using natural splines [38] to account for possible non-linearity. We assessed the possibility that in the BMI-adjusted models, the independent secular increase in waist circumference varied by race-ethnicity or age by including various interaction terms in the regression models. Similar models, but with a linear term for survey year, were constructed to assess the trend in waist circumference from 1988–1994 or 1999–2000 through 2011–2012.

Of the 26,981 6- to 19-year-olds who had a weight and height measurements, 548 (2%) were missing information on waist circumference. The distributions of sex, race-ethnicity and BMI levels among these 548 children, however, did not differ from those of the other 26,433 children who had a measured waist circumference.

### Ethics Statement

The NHANES protocol, including the consent procedure for minors, was approved by the Research Ethics Review Board of the National Center for Health Statistics. Written consent was obtained for all adults (≥ 18 years of age) and written, parental permission was obtained for minors under the age of 18 years. Children aged 7 to 17 years were also asked to provide documented assent.

## Results

**Table 1**and **Fig 1**show mean levels of the body size measures and the prevalence of obesity in each survey from 1988–1994 through 2011–12 among 6- to 19-year-olds. Over this approximately 20-year period, the mean waist circumference increased by 3.7 cm among boys and by 6.0 cm among girls. In addition, the mean BMI-for-age z-score increased by 0.25 to 0.30 SDs, and the prevalence of obesity increased by about 7 (boys) and 10 (girls) percentage points. As shown in Fig 1, most of the BMI and waist circumference increases occurred between 1988–94 and 2003–04. Over the approximately 20-year period, the mean BMI of boys increased by 5.7% (from 20.1 to 21.2 kg/m^2^) and the mean waist circumference increased by 5.3% (from 70.6 to 74.3 cm), while among girls, the percentage changes were 7.7% (BMI) and 8.7% (waist circumference). These similarities reflect the very strong cross-sectional correlation (r = 0.95) between BMI and waist circumference.

**Fig 1:**
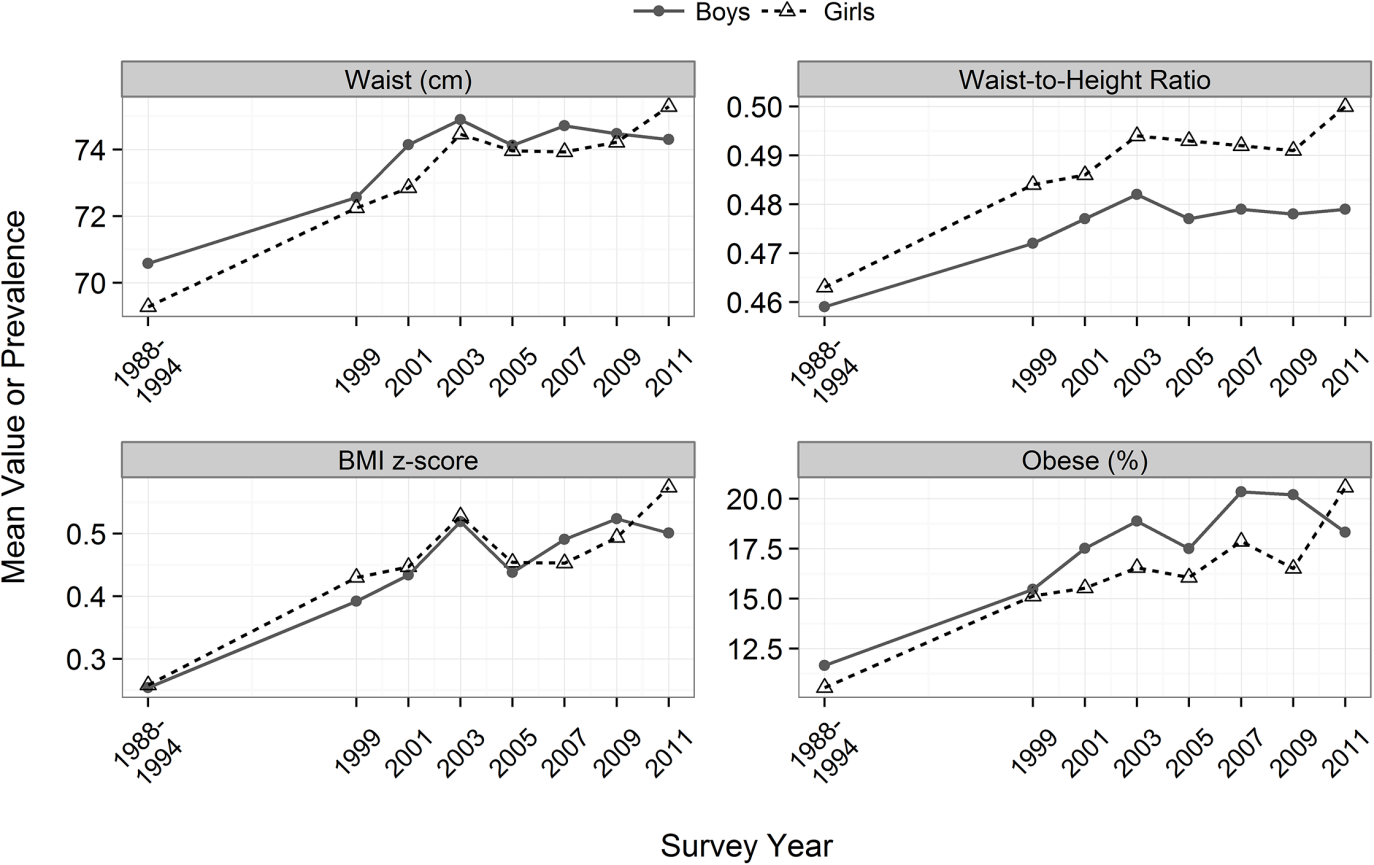
Mean levels of waist circumference, WHtR, BMI-for-age z-score and obesity among 6- to 19-year-olds from 1988–94 through 2011–12. Boys are represented by the solid, grey line.

**Table 1:** Mean levels (or prevalence) of various measures of body size among 6- to 19-year-olds from 1988–94 through 2011–12.

Sex	Year	Sample Size	Waist (cm)	Waist/Height	BMI(kg/m^2^)	BMI-for-age Z-Score	Prevalence of Obesity (95% CI)	Prevalence of Severe Obesity (95% CI)
Boys	1988–1994	3106	70.6 ± 0.4^a^	0.459 ± 0.002	20.1 ± 0.1	0.25 ± 0.03	11.7 (9.8, 13.7)^b^	3.2 (2.1, 4.7)
	1999–2000	1684	72.6 ± 0.9	0.472 ± 0.004	20.7 ± 0.3	0.39 ± 0.07	15.5 (12.7, 18.5)	4.5 (2.6, 7.0)
	2001–02	1735	74.1 ± 0.4	0.477 ± 0.002	21.1 ± 0.1	0.43 ± 0.04	17.5 (15.2, 20.0)	6.4 (5.0, 8.0)
	2003–04	1570	74.9 ± 0.8	0.482 ± 0.004	21.3 ± 0.3	0.52 ± 0.05	18.9 (15.4, 22.8)	5.6 (3.6, 8.2)
	2005–06	1615	74.1 ± 0.8	0.477 ± 0.003	21.1 ± 0.3	0.44 ± 0.06	17.5 (13.5, 22.1)	5.7 (3.7, 8.3)
	2007–08	1212	74.7 ± 0.7	0.479 ± 0.004	21.4 ± 0.3	0.49 ± 0.06	20.3 (17.0, 24.0)	6.4 (4.3, 9.1)
	2009–10	1264	74.5 ± 0.7	0.478 ± 0.003	21.5 ± 0.3	0.52 ± 0.04	20.2 (17.0, 23.8)	7.4 (5.0, 10.6)
	2011–12	1235	74.3 ± 0.6	0.479 ± 0.003	21.2 ± 0.2	0.50 ± 0.05	18.3 (14.9, 22.2)	6.6 (4.1, 9.8)
Girls	1988–1994	3165	69.3 ± 0.5	0.463 ± 0.002	20.3 ± 0.2	0.26 ± 0.04	10.5 (8.9, 12.4)	2.9 (1.9, 4.3)
	1999–2000	1589	72.2 ± 0.6	0.484 ± 0.003	21.0 ± 0.2	0.43 ± 0.04	15.1 (12.7, 17.9)	4.1 (2.8, 5.8)
	2001–02	1710	72.8 ± 0.7	0.486 ± 0.004	21.1 ± 0.3	0.45 ± 0.05	15.5 (12.2, 19.3)	4.6 (3.1, 6.4)
	2003–04	1501	74.5 ± 0.7	0.494 ± 0.004	21.5 ± 0.3	0.53 ± 0.05	16.5 (13.5, 19.9)	5.1 (3.6, 6.9)
	2005–06	1582	74.0 ± 0.8	0.493 ± 0.004	21.3 ± 0.3	0.45 ± 0.04	16.1 (12.1, 20.7)	5.5 (3.7, 7.8)
	2007–08	1126	73.9 ± 0.8	0.492 ± 0.004	21.4 ± 0.2	0.45 ± 0.05	17.9 (14.6, 21.5)	5.1 (3.3, 7.4)
	2009–10	1161	74.2 ± 0.5	0.491 ± 0.002	21.5 ± 0.2	0.49 ± 0.04	16.5 (14.9, 18.2)	5.7 (4.2, 7.6).
	2011–12	1178	75.3 ± 0.8	0.500 ± 0.005	21.9 ± 0.3	0.57 ± 0.06	20.6 (17.7, 23.7)	6.7 (4.7, 9.2)

^**a**^ Values are mean ± SE or prevalence (95% CI)

^**b**^ Based on a BMI-for-age ≥ 95th percentile of the 2000 CDC Growth Charts (reference #31) or a BMI≥ 30 kg/m^2^

**Fig 2:**
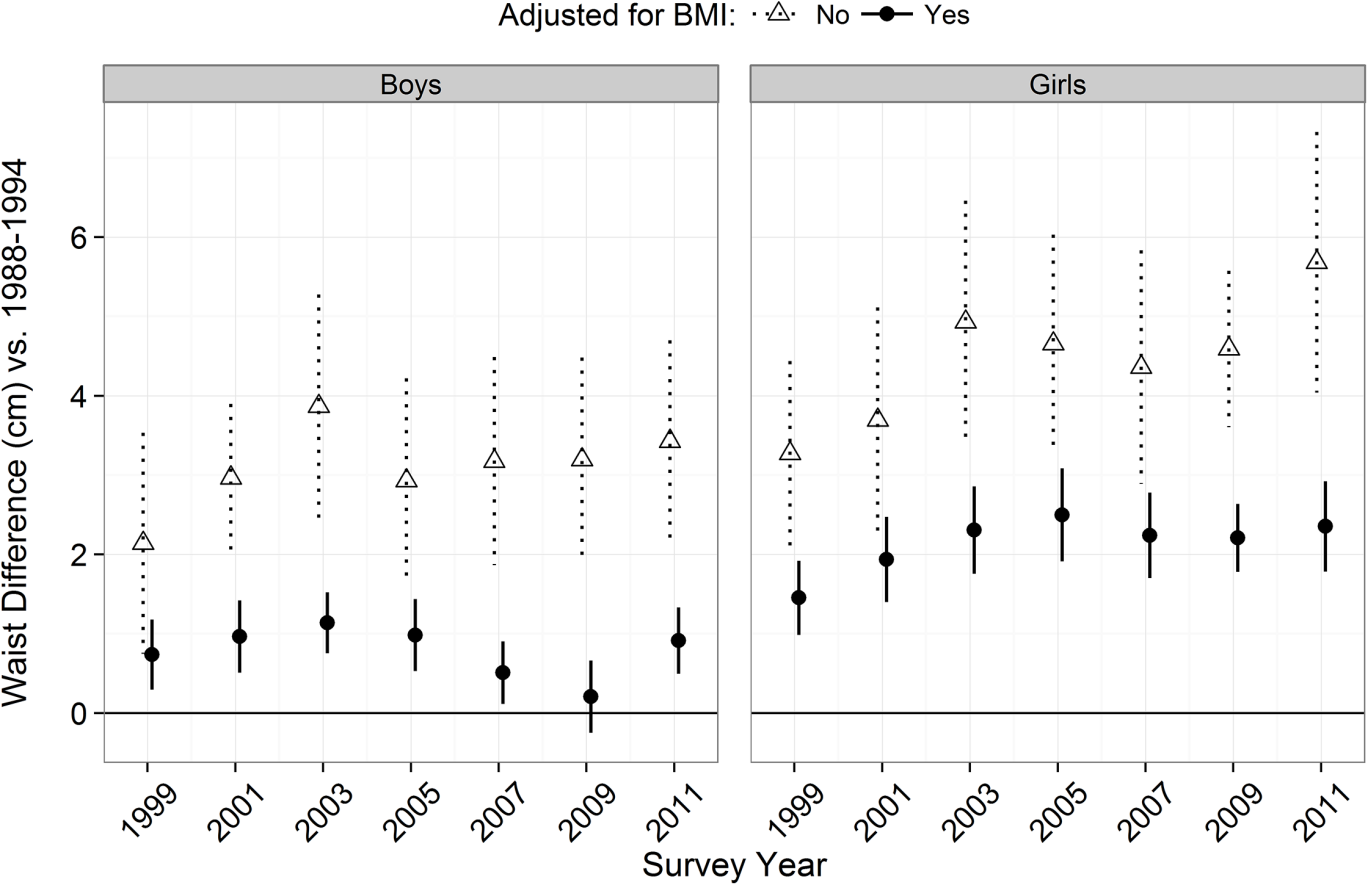
Regression coefficients and CIs for the difference in the mean waist circumference between 1988–94 and each subsequent survey cycle. Estimates were based on a regression model that included survey as a categorical level with 1988–94 as the reference category. The open triangles are estimates from models that included race and age, while the black circles are estimates from models that included BMI as an additional predictor. Age and BMI were modeled using natural splines with 3 knots. The 95% CIs for each estimate are also shown.

**Fig 2**shows the mean and 95% CI for the race- and age-adjusted differences in waist circumference (triangles and dotted CIs), between children in 1988–1994 (referent category in the regression models) and those in each subsequent survey. The mean waist circumference was substantially higher in 2003–04 than in 1988–94 (mean differences: +4 cm, boys; +5 cm, girls), but remained fairly stable subsequently. Including BMI as an additional covariate in these regression models (solid circles and lines) greatly reduced the differences among both boys and girls. Based on these BMI-adjusted regression models, the estimated difference in waist circumference between levels in 1988–94 and those in each subsequent survey ranged from 0.2 cm (2009–10) to 1.1 cm (2003–04) among boys and from 1.5 cm (1999–2000) to 2.5 cm (2005–06) among girls. Additional analyses indicated that adjustment for weight, rather than for BMI, yielded very similar results (data not shown).

As assessed by the areas between the x = 0 line and lines connecting either the unadjusted or adjusted estimates in **Fig 2**, we estimated that controlling for BMI accounted for 76% of the increase in mean waist circumferences among boys during this period. (The area under the curve, obtained by integration, was 38 cm × year based on the models without BMI, while the BMI-adjusted area was 9 cm × year.) The comparable reduction among girls was 51%.

Additional analyses that approximated the changes in waist circumference by a linear trend (**Table 2**) also indicated that controlling BMI greatly reduced the secular increases. Among boys, including BMI as a covariate reduced the increase in waist circumference from 1988–94 from 1.6 to 0.2 cm per decade (both estimates were statistically significant at the 0.05 level). Among girls, the corresponding reduction was from 2.5 to 1.1 cm per decade. Although the pattern was somewhat similar in analyses that examined the trend from 1999–2000 through 2011–12 (final column), even without adjustment for BMI, the increase in the waist circumference of boys during this period was not statistically significant. In contrast, the independent increase in waist circumference among girls between 1999–2000 and 2011–12 was statistically significant (β = +0.6 cm per decade, p = 0.003). The categorization of year (Table 2, bottom) in the regression models confirmed these findings. Controlling for BMI reduced the observed increase in waist circumference from 3.5 cm to 1.0 cm among boys and from 5.8 cm to 2.4 cm among girls. There was also an interaction between and waist circumference among girls (p < 0.01), but not boys, with the magnitude of the BMI-independent increase in waist circumference increasing with age (data not shown).

**Table 2:** Change in waist circumference (cm) among 6- to 19-year-olds.

			Secular Change Through 2011–12	
Classification of Year	Sex	Adjustment for BMI^a^	Starting in 1988–1994	Starting in 1999–2000
Linear Trend^b^	Boys	No	1.6 (1.0, 2.1)	0.6 (-0.4, 1.7)
		Yes	0.2 (0.02, 0.4)	-0.3 (-0.6, 0.0)
	Girls	No	2.5 (1.9, 3.1)	1.5 (0.4, 2.6)
		Yes	1.1 (0.9, 1.3)	0.6 (0.2, 0.9)
Categorical^c^	Boys	No	3.5 (2.2, 4.8)	1.2 (-0.5, 2.9)
		Yes	1.0 (0.6, 1.4)	0.2 (-0.3, 0.6)
	Girls	No	5.8 (4.1, 7.5)	2.4 (0.7, 4.1)
		Yes	2.4 (1.9, 3.0)	0.9 (0.4, 1.4)

^**a**^ Both models (without and with BMI as a covariate) control for race and age

^**b**^ Linear trends estimates are change in waist circumference per 10 years for the period starting in either 1988–94 or in 1999–2000 and ending in 2011–12. 95% CIs are shown in parentheses.

^**c**^ Categorical estimates of waist circumference represent the difference in waist circumference between levels in 1988–94 or 1999–2000 and levels in 2011–12. 95% CIs are shown in parentheses.

**Fig 3:**
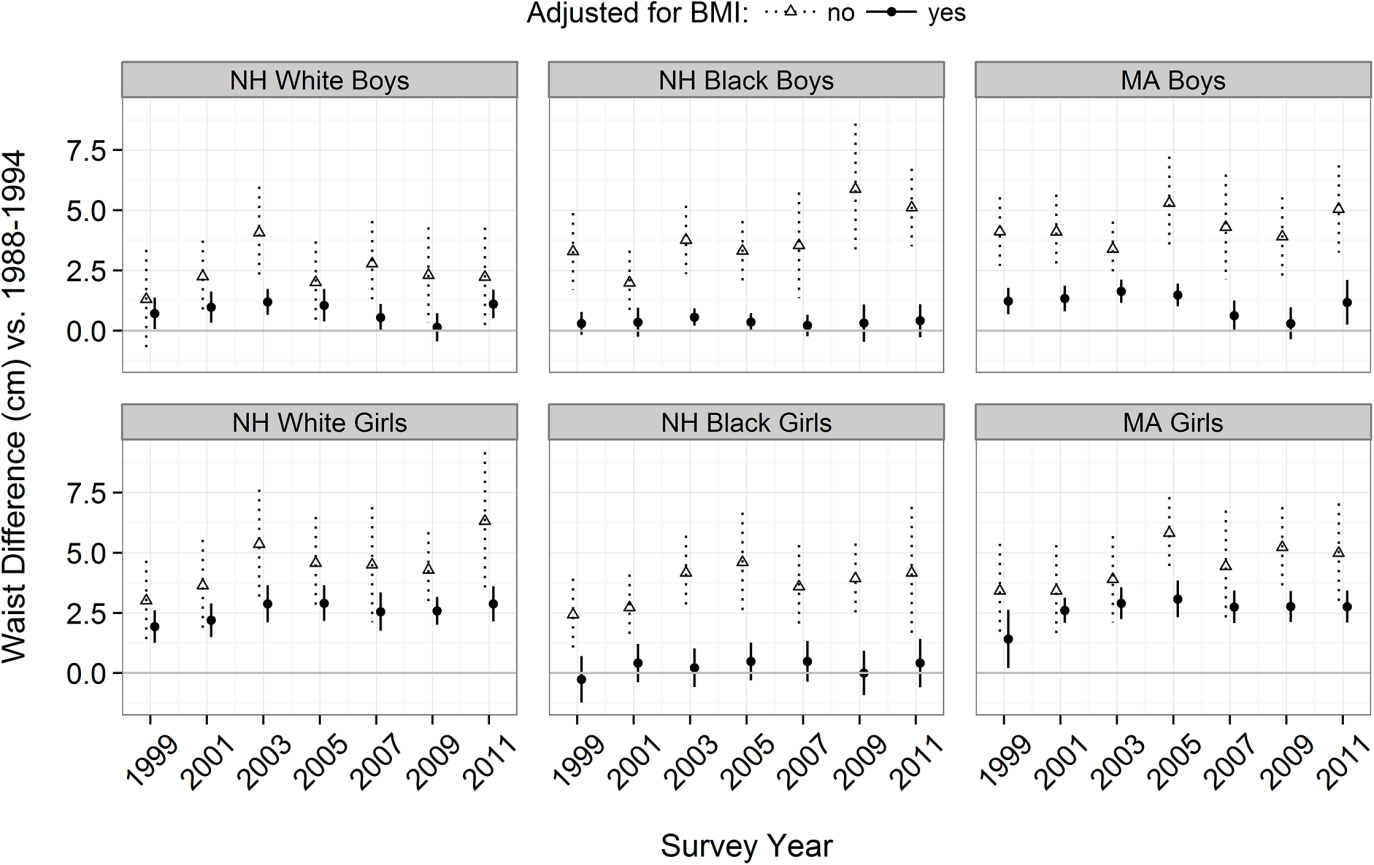
Regression coefficients showing the difference in mean waist circumference between 1988–94 and each subsequent survey by sex (boys, top row) and race-ethnicity (columns). The open triangles represent estimates that are adjusted only for age, and the black circles are estimates from models that adjust for both age and BMI.

Additional analyses indicated that the BMI-adjusted waist circumference changes varied by race-ethnicity among both boys (p < 0.01 for interaction term) and girls (p < 0.001). **Fig 3**shows differences in waist circumference in each survey cycle relative to children in 1988–94 for each race-ethnicity among boys (top row) and girls (bottom). Before adjustment for BMI (open triangles), the estimated waist circumference differences among NH black boys and Mexican-American boys were generally larger than those among white boys, but there were no consistent race-ethnicity difference in waist circumference among girls. However, adjustment for BMI greatly reduced the waist circumference increases among black children, with the mean increase decreasing from 3.8 cm (adjusted for age) to 0.4 (age- and BMI-adjusted) among black boys and from 3.6 cm to 0.35 cm among black girls over the 7 cycles. Overall, adjustment for BMI reduced the secular increases in waist circumference by about 90% among NH black boys and NH black girls, but by only 70% (boys) and 40% (girls) among NH white and Mexican-American children.

## Discussion

Our results indicate that although the mean waist circumference of 6- to 19-year-olds in the U.S. has increased by 4 cm (boys) to 6 cm (girls) since 1988–1994, much of this increase is due to factors that have led to increases in both BMI and waist circumference. In the current study, adjustment for BMI reduced the magnitude of the secular increase in waist circumference over the approximately 20-year period by about 75% among boys and 50% among girls. Furthermore, there has not been a statistically significant increase in the mean waist circumference of boys since 1999–2000. Additional analyses in the current study indicated that adjustment for weight, rather than BMI, resulted in very similar results concerning the independent increase in waist circumference.

In parallel with the secular increases in waist circumference that have been reported among adults [27,39], the waist circumference of children in various countries has increased over the last 20 years [19,20,23–25,40,41]. Although few of these studies attempted to determine if the change in waist circumference was independent of BMI, a recent review concluded that to some extent, there have been independent increases in waist circumference [20]. For example, some investigators have found increases in waist circumference but not in BMI [24] or that the proportional increases in waist circumference have been larger than those for BMI [25,42]. Furthermore, as assessed by skinfold thicknesses, it appears that the distribution of body fatness has become more centralized [43]. Several studies among adults have also concluded that the secular trends in waist circumference in the U.S. have been independent of BMI [27,44,22]. Few of these studies, however, have attempted to quantify how much of the secular increase in waist circumference was independent of the increase in BMI.

In contrast to these results, we found that the proportional changes in waist circumference were similar to those in BMI from 1988–94 through 2011–12, and that only ¼ (boys) and ½ (girls) of the secular increase in waist circumference was independent of BMI. The differences between our results and those of previous studies concerning the BMI-independent increases in waist circumference may be due to differences in time periods, statistical methods, and measurement techniques. For example, some studies [26,22] included data from the first National Health and Examination Survey (1959–1962), even though waist circumference was measured at a different location than in more recent NHANES studies [21,31] (http://www.cdc.gov/nchs/data/nhanes/nhes123/1003). Furthermore, although Walls et al. [27] emphasized the approximately 0.75 cm independent increase in waist circumference from 1988–94 through 2005–2006, there was no mention that BMI adjustment reduced this estimate by about 80%.

The race-ethnicity difference that we observed, with BMI accounting for about 90% of the secularly increase in the waist circumference of both NH black boys and NH black girls, was unexpected and the reasons for this interaction are uncertain. Over the 20-year period, NH black children showed a larger secular increase in BMI (~2 kg/m^**2**^) than did NH white and Mexican-American children (~1.0 to 1.3 kg/m^**2**^). Differences in the waist circumference trends across race-ethnicity groups were smaller, ranging from +4.6 cm (whites) to +5.4 cm (blacks and Mexican-Americans). As compared to white adults, blacks have less intra-abdominal fat than do whites [45] and we found that the mean, BMI-adjusted waist circumference was 3 cm lower among NH black children than among other children (data not shown). However, the proportion of abdominal fat that is in the subcutaneous region is higher among black adults than among whites and Hispanics [46], and this fat depot appears to be the most strongly associated with BMI [47]. If similar differences exist among children, this may explain why BMI adjustment had the largest effect on the waist circumference trends among NH black children.

Several of the results of the current study are similar to our recent findings among adults [21]. Although there were large secular increases in waist circumference since 1999–2000, with mean increases of 2 cm among men and 4 cm among women, controlling for BMI reduced this increase by approximately 90% among men and 20% among women. A recent analyses of secular trends in abdominal obesity from 2003–04 through 2011–12 among children and adolescents in NHANES [41] reported that there was no change in waist circumference since 2003–04, but this may not be the optimal starting point for an analysis of secular trends. As seen in Table 1 and Fig 1, levels of waist circumference and BMI-for-age appear to have been slightly higher in 2003–04 than in adjacent cycles.

Several possible reasons explanations have been proposed for the observed secular increases in the waist circumference of adults [27,22], including changes in energy intake and physical activity, the prevalence of endocrine disruptors, and treatments for depression [48] and obesity [49]. It is also possible that changes in drug treatment for attention-deficit/hyperactivity disorder (ADHD) [50] may play a role in these secular trends, but it is likely that this would influence trends in adult obesity more than child obesity [51,52]. A difficulty in assessing possible mechanisms is that the relation of these characteristics to obesity is likely to be much weaker than is the cross-sectional correlation (r>0.90) between BMI and waist circumference.

It is, however, possible that BMI-independent increases in waist circumference may simply reflect that fat mass is more strongly associated with waist circumference than with BMI. Although BMI and waist circumference are strongly correlated, there is some evidence [53] among adults that the correlation between longitudinal changes in these 2 measure of body size are weaker than are the cross-sectional correlations. For example, over a 5-year period, the correlation between weight change and waist circumference change was r ~ 0.70.

There are several limitations of the current study that should be considered in the interpretation of our results. Although about 2% of the children who had a measured BMI were missing data for waist circumference, the probability of missingness was not related to BMI and the exclusion of these children is unlikely to have biased our findings. It should be realized, however, that the measurement of waist circumference in NHANES is just above the iliac [31], a location that has been characterized as being difficult to accurately measure [54]. In contrast, other studies of children and adolescents have measured waist circumference midway between the lower rib and the top of the iliac crest [24,25,40,42]. The relatively small (N ≤ 1700) sex-specific sample sizes, combined with the multi-stage cluster design of NHANES, also resulted in the 2-year estimates being relatively imprecise. It should also be noted that the magnitudes of the secular increases in waist circumference among girls were increased with age (p < 0.01 for interaction) with 16- to 19-year-old girls, for example, showing more than a 3.0 cm (BMI-independent) increase over the approximately 20-year period.

Our results indicate that among children, levels of BMI and waist circumference are, to some extent, particularly among boys, responding similarly to various environmental and lifestyle characteristics that are causing levels of both measures to have increased since 1988–1994. These increases, however, appear to have plateaued or slowed in recent years. The very strong correlation between waist circumference and BMI makes it important to consider BMI-adjusted, rather than simply crude, change in levels of waist circumference over time. Possible reasons for the very large effects of BMI adjustment on the secular increase in waist circumference among NH black children should be further explored.

## Abbreviations

ADHD: attention-deficit/hyperactivity disorder
BMI: body mass index
CDC: Centers for Disease Control
NH: non-Hispanic
NHANES: National Health and Nutrition Examination Survey
WHtR: waist to height ratio
